# Dosimetric verification and quality assurance of running‐start‐stop (RSS) delivery in tomotherapy

**DOI:** 10.1120/jacmp.v16i6.5336

**Published:** 2015-11-08

**Authors:** Francis Kar‐ho Lee, Simon Kar‐yiu Chan, Ricky Ming‐chun Chau

**Affiliations:** ^1^ Medical Physics Division Department of Clinical Oncology, Queen Elizabeth Hospital Hong Kong SAR China

**Keywords:** tomotherapy, running‐start‐stop delivery, dynamic jaw

## Abstract

The purpose of this study was to evaluate the dosimetric profiles and delivery accuracy of running‐start‐stop (RSS) delivery in tomotherapy and to present initial quality assurance (QA) results on the accuracy of the dynamic jaw motion, dosimetric penumbrae of the RSS dynamic jaw and the static jaw were measured by radiographic films. Delivery accuracy of the RSS was evaluated by gamma analysis on film measurements of 12 phantom plans. Consistency in the performance of RSS was evaluated by QA procedures over the first nine months after the installation of the feature. These QA were devised to check: 1) positional accuracy of moving jaws; 2) consistency of relative radiation output collimated by discrete and continuously sweeping jaws; 3) consistency of field widths and profiles. In the longitudinal direction, the dose penumbra in RSS delivery was reduced from 17.3 mm to 10.2 mm for 2.5 cm jaw, and from 33.2 mm to 9.6 mm for 5 cm jaw. Gamma analysis on the twelve plans revealed that over 90% of the voxels in the proximity of the penumbra region satisfied the gamma criteria of 2% dose difference and 2 mm distance‐to‐agreement. The initial QA results during the first nine months after installation of the RSS are presented. Jaw motion was shown to be accurate with maximum encoder error less than 0.42 mm. The consistency of relative output for discrete and continuously sweeping jaws was within 1.2%. Longitudinal radiation profiles agreed to the reference profile with maximum gamma <1 and field width error <1.8%. With the same jaw width, RSS showed better dose penumbrae compared to those from static jaw delivery. The initial QA results on the accuracy of moving jaws, reproducibility of dosimetric output and profiles were satisfactory.

PACS number: 87.55.km

## I. INTRODUCTION

Running‐start‐stop (RSS) dynamic jaw delivery in helical tomotherapy is currently available. With this delivery mode, the jaws open and close around the superior and inferior end of the treatment target in an asymmetric sweeping motion that results in smaller dose penumbrae as compared to the conventional static jaw delivery. The back jaw opens from the smallest jaw width (1 cm) when it approaches the target, and while the front jaw approaches the end of the target, it closes to the smallest jaw width. With these characteristics, the RSS mode is available for the 2.5 cm and 5 cm jaw, but not for the 1 cm jaw. Previous reports showed dosimetry benefits, such as reduction in treatment time and improved longitudinal dose conformity, with the use of this feature.^1^, ^2^, ^3^ However, measurement of the improved dose penumbra, delivery accuracy, and the results of quality assurance (QA) demonstrating the accuracy of the jaw motion were yet to be studied. This report includes the results of a series of measurements to verify the delivery of the RSS. Initial QA results on the delivery accuracy of the RSS feature are also presented.

## II. MATERIALS AND METHODS

**Figure 1:**
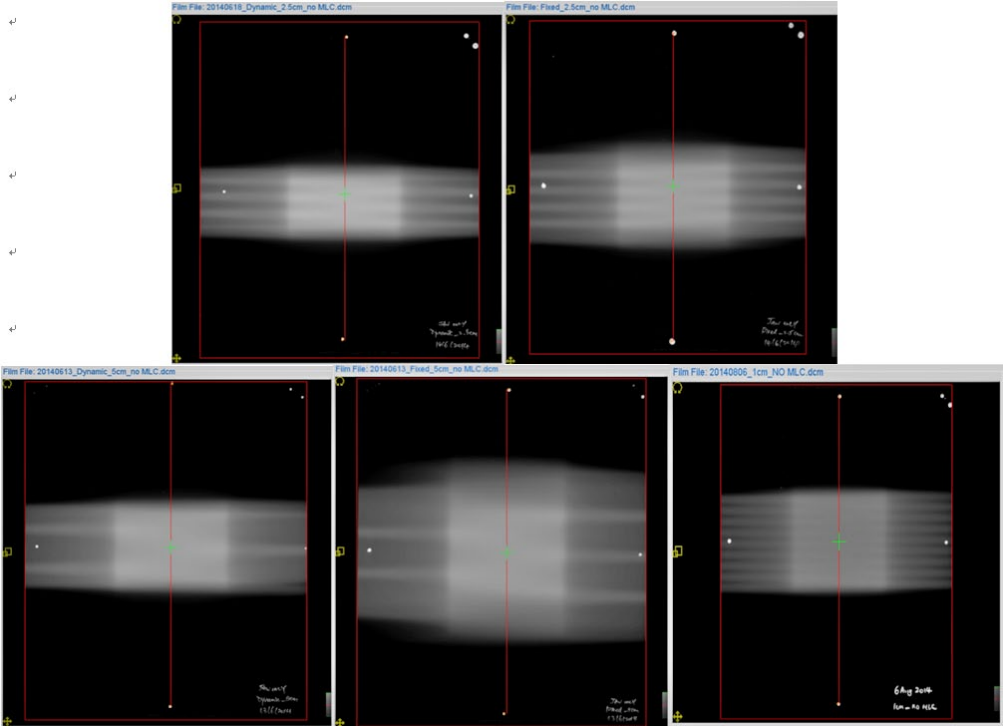
Films irradiated to measure the penumbra in the longitudinal profile. RSS with 2.5 cm jaw (upper left) and 5 cm jaw (lower left). Static jaw with 2.5 cm (upper right) and 5 cm jaw (lower middle). Static jaw with 1 cm (lower right).

The dosimetric penumbrae, as defined by the distance between 20% and 80% of the target dose in the superior–inferior direction, delivered by the RSS dynamic jaw and static jaw in a helical tomotherapy system (TomoHD, Accuray Inc., Sunnyvale, CA), were measured by radiographic films (EDR‐2, Carestream Health Inc., Rochester, NY). These films were positioned on the central coronal plane of a cylindrical Solid Water phantom. Helical delivery patterns were programmed with procedure creation tools and sinogram editor in five different settings (shown in Table 1), representing RSS and static jaw delivery for 2.5 cm and 5 cm jaws, and static jaw delivery for the 1 cm jaw. All multileaf collimators (MLCs) were static during the delivery, with the central 16 MLCs opened and the remaining MLCs closed. Films obtained from each of the five settings were developed with the standard method and recorded with a film digitizer (VXR‐12 DosimetryPRO Advantage, Vidar Systems Corp., Herndon, VA) in scanning software (Film Analyzer, Accuray Inc.). Films were calibrated by EDR‐2 films irradiated in the TomoTherapy System (Accuray Inc.) with square field sizes and at different levels of dose measured by an ionization chamber (A1SL, Standard Imaging, Middleton, WI). The profiles along the central longitudinal axis of the films were plotted and are shown in Fig. 1. The penumbrae on the back and front jaws were measured.

**Figure 2:**
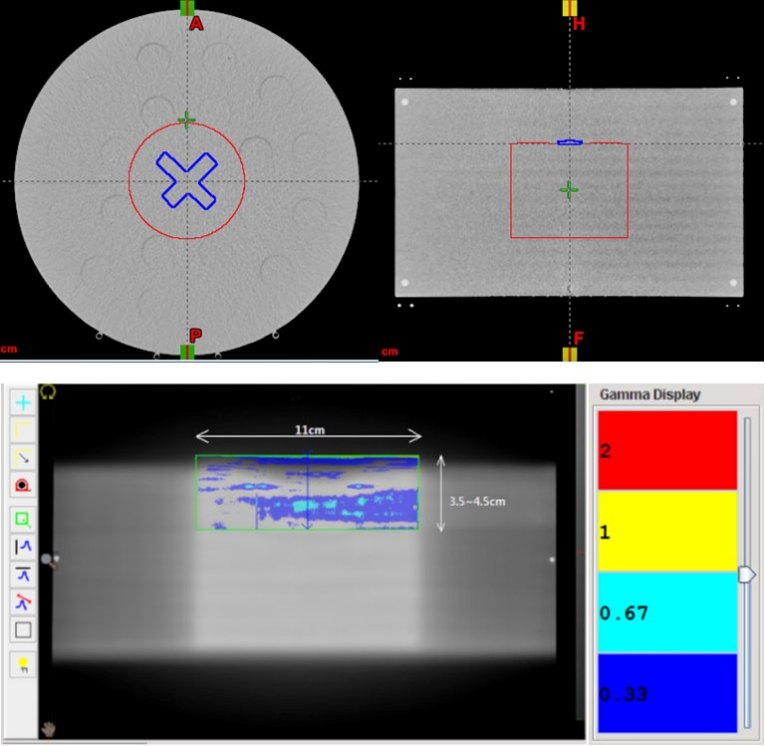
(Upper) Phantom contour (set B) with avoidance structure in blue and pseudotarget in red; (middle) region of interest for gamma analysis between the measured and the calculated dose; (lower) a plot of the dose measured and calculated dose profiles for this plan.

To evaluate the delivery accuracy of RSS, treatment plans were created with contours delineated on a cylindrical phantom (Fig. 2). The TomoTherapy Planning Workstation (Version 5.0.2.5, Accuray Inc., Sunnyvale, CA) used the collapsed cone algorithm for dose calculation, and the accuracy of this algorithm was shown to agree with Monte Carlo simulation and measurement in homogeneous^(4)^ and heterogeneous phantoms.^(5)^ Three sets of contours were designed to simulate scenarios of increasing dose modulation, with an avoidance structure drawn intruding on the superior border of a pseudotreatment target. The depth of intrusion enabled MLC modulation to create increased rapid dose falloff between the avoidance structure and the target. In contour set A, the avoidance structure was located adjacent to the target; in contour set B, the avoidance structure extended 1 mm longitudinally into the target; in contour set C, the avoidance structure extended 2 mm longitudinally into the target. Twelve treatment plans were created, based on combinations of different structure sets, jaw sizes, and delivery modes, shown in Table 2. Films were positioned on the central coronal plane of the phantom to measure the dose distribution. After developing and scanning, the films were registered to the planned dose distribution by the use of pinprick markers on the vendor‐supplied QA software. Gamma analysis according to the algorithm proposed by Low et al.,^(6)^ with the criteria of 2% dose difference and 2 mm distance‐to‐agreement, was performed over the region that encompassed the entire penumbra. A region of interest (ROI) of 3.5 cm in the longitudinal direction was sufficient to cover the whole penumbra for 2.5 cm jaw, while a larger ROI (4.5 cm) was used for 5 cm jaw due to the larger penumbra in the static jaw mode. Laterally, the ROI encompassed the entire width of the pseudo‐target. The size and location of the ROI is shown in Fig. 2.

**Table 1:** Parameters used in the helical delivery for the film measurement of dosimetric penumbra

	*Setting 1*	*Setting 2*	*Setting 3*	*Setting 4*	*Setting 5*
Delivery jaw mode	Static	RSS	Static	RSS	Static
Jaw width (cm)	2.5	2.5	5	5	1
No. of projections	255	255	169	169	450
Projections per sec	3.4	3.4	3.4	3.4	3.4
No. of gantry rotations	5	5	3.31	3.31	8.82
Couch travel distance (cm)	7.5	7.5	11.6	11.6	8.82
Procedure time (sec)	75	75	49.71	49.71	132.35

**Table 2:** Gamma analysis results of the 12 QA plans for evaluation of treatment delivery accuracy in RSS and static jaw delivery

*Contour Set*	*Mode*	*Jaw Width*	*% voxel (3%, 3 mm, gamma* <1)	*% voxel (2%, 2 mm, gamma* <1)
A	Static	2.5 cm	99.0	94.0
A	RSS	2.5 cm	98.5	93.8
A	Static	5 cm	99.0	96.8
A	RSS	5 cm	99.0	97.8
B	Static	2.5 cm	99.0	98.0
B	RSS	2.5 cm	99.0	98.8
B	Static	5 cm	99.0	98.6
B	RSS	5 cm	99.0	97.6
C	Static	2.5 cm	98.5	90.8
C	RSS	2.5 cm	99.0	95.0
C	Static	5 cm	99.0	91.0
C	RSS	5 cm	98.5	94.0

The consistency and accuracy in the RSS delivery were evaluated by monthly QA procedures during the first nine months after installation of the feature. These QAs were scheduled at a frequency suggested by the vendor and were also referenced to similar requirements on static jaw delivery according to the AAPM TG‐148 report.^(7)^ The following were checked in the QA:

Positional accuracy of moving jaws: Jaw positions were monitored by encoders that detected the jaw position at intervals of 2 milliseconds during a "sweeping‐jaw" QA procedure. The maximum error in each procedure, expressed in millimeters projected to the isocenter for the front and back jaws, was recorded.
2. Consistency of relative jaw fluence output factor in discrete and continuously sweeping jaws: The relative radiation output received by a 1.91 cc cylindrical ionization chamber (A17, Standard Imaging Inc.) positioned in a rectangular Solid Water phantom, under discrete and continuous sweeping motion of different jaw widths (1 cm, 2 cm, and 2.5 cm), were obtained and compared against the reference data. Deviation from the reference revealed motion or positional error of the jaws.
3. Consistency of field widths and profiles: Profiles were measured by a 0.053 cc cylindrical ionization chamber (A1SL, Standard Imaging Inc.) positioned on the moving couch under different symmetric and asymmetric jaw widths (1 cm, 2 cm, 2.5 cm, and 5 cm). The profiles were scanned and recorded in step sizes of approximately 0.19 mm. The correspondence between the reference profiles and the measured profiles were evaluated with gamma analysis. The effective field widths were also compared.

## III. RESULTS

Films obtained from the static and RSS delivery for the different jaw widths were scanned and are shown in Fig. 1. The penumbrae measured along the central longitudinal axis for the front and back jaws are shown in Table 3. For the same jaw width, the penumbra was substantially reduced with the use of RSS delivery, although still larger than that from a 1 cm jaw.

The gamma analysis on the films for all 12 treatment plans, using 3%/3 mm and 2%/2 mm criteria, satisfied our acceptance criteria for plan QA, of which over 90% of the voxels had gamma <1. The results are shown in Table 2.

The QA results in the first nine months after installation of the RSS are shown in Table 4. In general, the criteria as described in AAPM TG‐148 on static jaw were fulfilled. Satisfactory QA results were recorded and no preventive maintenance action was initiated or suggested.

**Table 3:** Comparison of measured penumbrae between RSS and static jaw

	*Front Jaw Penumbra*		*Back Jaw Penumbra*	
	*RSS*	*Static*	*RSS*	*Static*
1 cm jaw	N/A	7.5 mm	N/A	7.4 mm
2.5 cm jaw	10.0 mm	17.5 mm	10.3 mm	17.1 mm
5 cm jaw	9.6 mm	33.2 mm	9.6 mm	33.2 mm

**Table 4:** Summary of QA result for dosimetric and mechanical accuracy of moving jaws

*Positional Error (mm) of Jaw Detected by Encoders*				
		*Front Jaw*		*Back Jaw*
Mean Error		0.140		−0.083
SD		0.162		0.222
Maximum Error		0.412		0.323
*Jaw Output Factor (Discrete Jaw Sweep)*				
*(% error as compared to reference output)*				
	*1.0 cm Jaw*	*2.0 cm Jaw*	*2.5 cm Jaw*	*5.0 cm Jaw* ^a^
Mean Error	0.13	0.05	0.13	NA
SD	0.67	0.27	0.14	NA
Maximum Error	1.1	0.38	0.29	NA
*Jaw Output Factor (Continuous Jaw Sweep)*				
*(% error as compared to reference output)*				
Mean Error	0.58	0.24	0.14	NA
SD	0.35	0.09	0.07	NA
Maximum Error	1.20	0.38	0.22	NA
*Maximum Gamma from Analysis Between Reference Profile and Measured Profile* ^b^				
Mean Gamma	0.343	0.355	0.342	0.508
SD	0.172	0.111	0.120	0.136
Maximum Gamma	0.977	0.617	0.629	0.723
*Field Width % Errors*				
Mean Error	0.237	−0.027	−0.232	−0.238
SD	0.752	0.464	0.297	0.189
Maximum Error	1.765	0.879	−0.344	−0.495

a Sweeping jaw patterns were not implemented for 5 cm jaw since it is the largest jaw opening possible for the TomoTherapy system.

b Gamma analysis criteria: 2% dose difference, DTA = 1% of jaw width for symmetric jaw setting, 0.5 mm for asymmetric jaw setting.

## IV. DISCUSSION

The RSS delivery resulted in rapid dose falloff in the longitudinal direction and better sparing of normal tissues beyond the treatment target. A substantial improvement in dose penumbra was demonstrated in this report. In either the 2.5 cm or 5 cm jaw width, the dose penumbrae were reduced to about 10 mm by the use of the RSS feature. This was larger than the penumbra of the traditional 1 cm jaw, which was about 7.5 mm. One reason for this was a characteristic of the RSS delivery: when the superior end of the target approached the beam, the back jaw gradually opened from the minimum jaw width to the preselected jaw width. The combined dosimetric effect created a beam divergence that was larger than that given by the 1 cm jaw.

The dosimetric accuracy of the RSS delivery in helical tomotherapy was evaluated by Sterpin^(8)^ using Monte Carlo simulation. In his study, the penumbrae of the longitudinal profile, calculated by the collapsed cone algorithm in a homogeneous phantom, agreed with the simulation within 0.6 mm. This echoes our gamma analysis results of the 12 treatment plans, of which 90% of the voxels satisfied the 2%/2 mm criteria. It has to be mentioned that film QA was prone to error by human setup and film processing. In our experience, the setup error using the pinprick marker to register the films and the isodose distribution was not more than 1.5 mm. Moreover, the films were developed and calibrated in a well‐controlled process. These minimized the measurement error and the 2%/2 mm criterion in the gamma analysis was considered appropriate.

This report also evaluated the dosimetric and mechanical accuracy of the RSS delivery. The RSS delivery was realized after the upgrade in jaw actuators, jaw linear encoder scales, jaw encoder read‐heads, and a new beam model that characterized the dosimetric behavior of the moving jaws. Delivery accuracy relied heavily on accurate movement of the jaws.

Jaw movement was evaluated in the QA results during the first nine months after installation of the RSS feature. Jaw motion was shown to be accurate, with the maximum encoder error <0.42 mm. Consistency of relative fluence output factors for discrete and continuously sweeping jaws were within 1.1%. Longitudinal radiation profiles were shown to be consistent with the reference profile, which passed the gamma analysis with maximum gamma <1, and maximum field width error <1.8%. Despite that no international guidelines were available for the frequency and tolerance of dynamic jaw QA, the requirement in AAPM TG‐148^(7)^ on static jaw delivery was referenced. The tolerances in output consistency (2%) and Y jaw positional accuracy (0.5 mm) were satisfied. However, the tolerance in field width (1%) was exceeded in one QA episode for the 1 cm jaw. The result was investigated and adjustment would be initiated upon persistent trend.

It is worth mentioning that the jaw actuator was replaced in the sixth month after installation of the RSS. After this, there was a slight increase, of less than 1% in magnitude, in the relative jaw fluence output factor and the jaw width. The trend was monitored before an adjustment was justified. On the other hand, the patient‐specific QA for individual plans after actuator replacement did not reveal this trend, suggesting that the machine QA used in this report might be more sensitive to changes in machine performance. Overall, a stringent QA was necessary to ensure consistent performance of the dynamic jaws, especially after replacement of relevant parts, such as the jaw actuator or encoder. Monthly QA on the dosimetric and mechanical accuracy of the jaws, with tolerances recommended on static jaws in TG‐148, seemed reasonable and practical according to our experience.

Previous reports^1^, ^2^, ^3^ on the dosimetric benefit of RSS emphasized the reduction in treatment time. The rationale was that RSS improved dose falloff at the superior and inferior end of the target, thus enabling the user to select a wide jaw size with shorter delivery time without sacrificing plan quality. This was true especially in cases such as treatment of thoracic lesions. A wider jaw size significantly reduced treatment delivery time by 20%–50%, while the sparing of nearby parallel organs such as lungs or liver was not compromised. However, this was not always the case, since the existing RSS feature improved the dose penumbra only at the start and at the end of the treatment target. Dose penumbra inside the treatment target was not affected, and therefore dose conformity or sparing of organs at risk enclosed by the treatment target was not improved. An example of this was the treatment of head and neck lesions where organs such as auditory organs or parotid glands were engulfed by the treatment target. Despite this, the use of RSS in the treatment of head and neck lesions was still favorable in that dose to optic organs and lungs could be reduced by as much as 50% according to our clinical experience. In future, jaw motion throughout the whole treatment length, with flexible dynamic modulation of jaw widths, as described in Chen et al.,^(9)^ could bring further dosimetry advantage. With our satisfactory results in the delivery accuracy of the dynamic jaw, there was increased confidence in the practicality of moving‐jaw delivery in tomotherapy.

## V. CONCLUSIONS

The improved penumbra and delivery accuracy of RSS were shown. The initial QA results of dynamic jaws, with regard to the positional accuracy, reproducibility of the dose output, and profiles of dynamic jaws, were satisfactory. The reliability of this new treatment mode was demonstrated in our machine.

## Supporting information

Supplementary Material
