# Supplementary material for: Dosimetric verification and quality assurance of running‐start‐stop (RSS) delivery in tomotherapy
Source: J Appl Clin Med Phys. 2015 Nov 8;16(6):23–9. doi: 10.1120/jacmp.v16i6.5336 (PMC5691007; doi:10.1120/jacmp.v16i6.5336)
Supplement: Supplementary file 1 — Supplementary Material [file ACM2-16-023-s001.docx]

Title: Dosimetric verification and quality assurance of Running-Start-Stop (RSS) delivery in TomoTherapy

Authors:

Francis Kar-ho Lee^1^,

Simon Kar-yiu Chan^1^,

Ricky Ming-chun Chau^1^

^1^Medical Physics Division, Department of Clinical Oncology, Queen Elizabeth Hospital, Hong Kong SAR, China

Correspondent author:

Name: Francis Kar-ho Lee

Address: Room 1214, Block R, Queen Elizabeth Hospital, 30, Gascoigne Road, Kowloon, Hong Kong

Email: [leekarhof@hotmail.com](mailto:leekarhof@hotmail.com)
